# Phytocannabinoids and bone health

**DOI:** 10.1007/s11914-026-00982-1

**Published:** 2026-09-08

**Authors:** Hayley Palmer, Diana E. Roopchand

**Affiliations:** Department of Food Science and New Jersey Institute for Food, Nutrition and Health, School of Environmental and Biological Sciences, The State University of New Jersey, RutgersNew Brunswick, NJ 08901 USA

**Keywords:** Cannabinoids, Cannabidiol, Cannabis, Bone remodeling

## Abstract

**Purpose of the Review:**

Phytocannabinoids have received attention for their therapeutic properties, however, it is unclear how they affect bone homeostasis. Additionally, increased use of cannabis and hemp-derived products have raised questions about their impact on musculoskeletal health. This review summarizes recent studies that investigate the impact of phytocannabinoids on bone.

**Recent Findings:**

The most well studied phytocannabinoid in relation to bone health is cannabidiol (CBD). In vitro data suggests CBD inhibits osteoclasts and promotes osteoblast activity. Rodent studies have demonstrated CBD may be useful for managing bone loss associated with osteoporosis and periodontitis, as well as reducing fracture pain and promoting healing. Recent studies suggest other phytocannabinoid isolates and extracts containing multiple phytocannabinoids have varying effects on bone. The first clinical study in this area reported that a medical cannabis product reduced markers of bone turnover in healthy adults.

**Summary:**

Cannabinoids bind to receptors in the endocannabinoid system which is involved in regulation of bone metabolism. Phytocannabinoids may improve bone health; however, additional work is required to determine factors such as the optimal dose, timing, and route of administration.

## Introduction

**Fig. 1:**
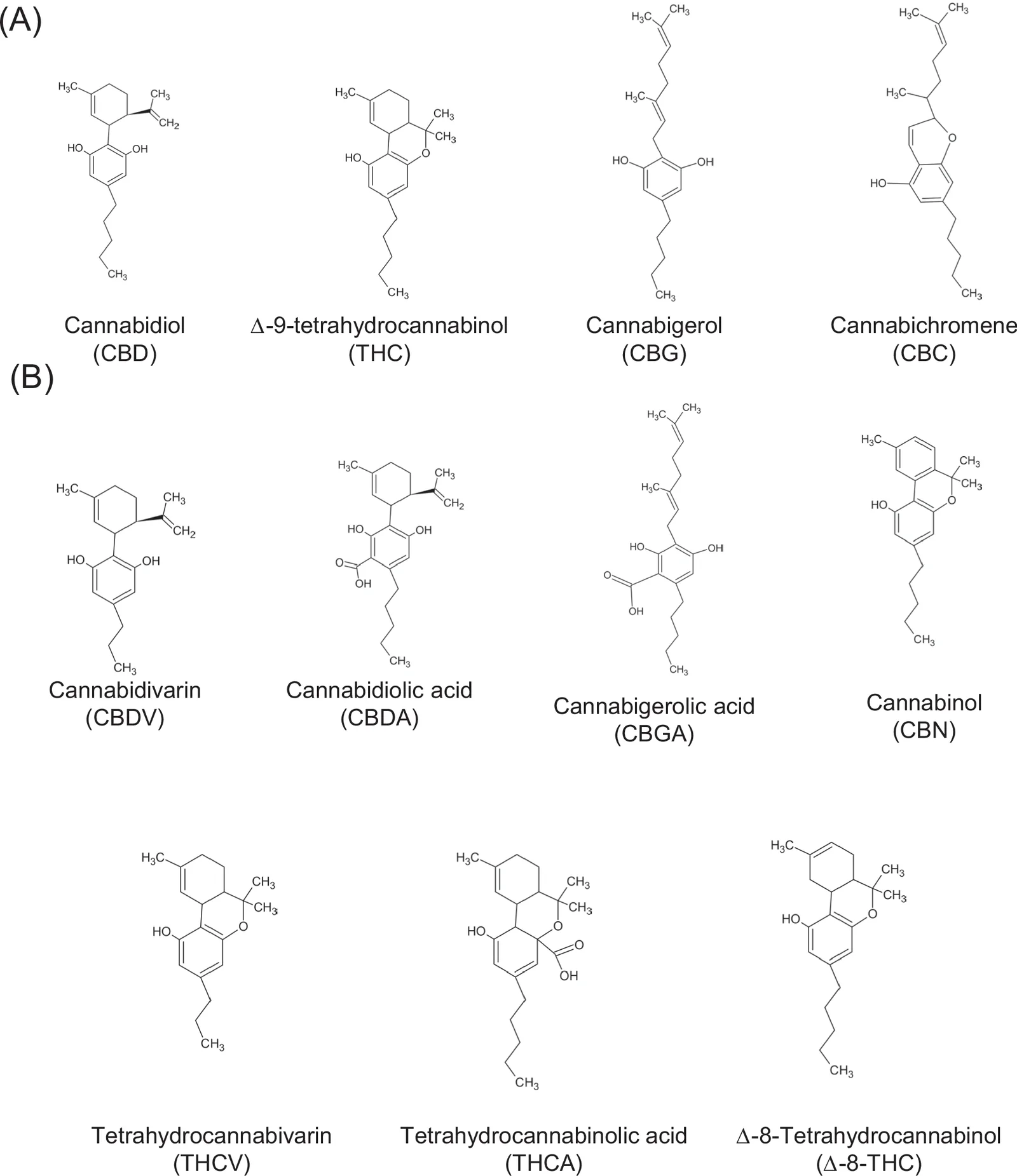
(**A**) Phytocannabinoids that have been studied in relation to bone. (**B**) Other phytocannabinoids found in cannabis

Due to their diverse pharmacological properties, phytocannabinoids may have therapeutic value for bone-related disorders. Cannabidiol (CBD), is the primary non-intoxicating phytocannabinoid derived from *Cannabis sativa* L. In the United States, hemp is currently defined as *Cannabis sativa* L. products containing less than 0.3% Δ−9-tetrahydrocannabidiol (THC), the main intoxicating compound. There are currently no FDA-approved cannabinoid-based drugs for treatment of bone diseases. Epidiolex® is an oral solution of CBD isolate that is FDA-approved for treating seizures associated with Lennox-Gastaut syndrome (LGS), Dravet syndrome, or tuberous sclerosis complex (TSC) in children over one year of age and adults [1, 2]. Dronabinol (Marinol®) is an orally administered form of synthetic THC that is FDA-approved for management of chemotherapy-induced nausea and vomiting [3, 4]. Nabiximols (Sativex®), an oral mucosal spray containing equal parts CBD and THC, has been approved for multiple sclerosis-induced muscle spasms and stiffness in Canada, the United Kingdom, and parts of Europe, but not the United States [5]. Though CBD and THC are the most well studied phytocannabinoids, over 100 minor phytocannabinoids have been identified in cannabis [6, 7]. Structures of CBD, THC, and some minor phytocannabinoids are shown in Fig. 1. There is growing research interest in these minor phytocannabinoids, particularly those with non-intoxicating effects such as cannabigerol (CBG), cannabichromene (CBC), Δ9-tetrahydrocannabivarin (Δ9-THCV), and cannabidivarin (CBDV) [8]. Cannabinoids bind to receptors in the endocannabinoid system (ECS), which regulate many physiological processes including bone remodeling [8, 9]. This review will summarize recent findings on how phytocannabinoids affect bone.

### The Endocannabinoid System Regulates Bone Metabolism

The ECS is composed of endocannabinoids (EC), receptors, and enzymes. N-arachidonoylethanolamide (AEA) and 2-arachidonoylglycerol (2-AG) are endogenous cannabinoids that regulate bone homeostasis [9, 10]. The primary cannabinoid receptors, CB1 and CB2, are G-protein coupled receptors involved in bone metabolism as evidenced by experiments in murine knock-out models [9, 11]. CB1^−/−^ mice showed increased peak bone mass and reduced bone resorption but developed age-related osteoporosis [12]. CB1^−/−^ mice also showed increased bone mineral density (BMD) and protection from bone loss in ovariectomized (OVX) mice [13]. These findings suggest that CB1 activates osteoclast activity but is required for bone marrow adipocyte and osteoblast differentiation. CB2 has a protective role in bone metabolism and was shown to promote osteoblast activity and reduce bone resorption [13]. Male and female CB2^−/−^ mice showed increased trabecular bone loss, cortical expansion, and high bone turnover [14]. The variable effects of CBD, THC, and other minor cannabinoids on bone are partially attributed to their differing binding affinities for CB1 and CB2. THC is a partial agonist for CB1 and CB2; in contrast, CBD has lower binding affinity and is an allosteric modulator of these receptors [15–20]. CBD reversed the LPS-induced downregulation of osteogenic markers through activation of CB2-dependent p38 MAPK signaling in bone marrow mesenchymal stem cells (BMSCs), suggesting CB2 can promote osteogenesis through CB2 activation [21]. CBD may also inhibit bone resorption through antagonism of GRP55, a putative cannabinoid receptor that stimulates osteoclast function [22, 23]. CBD increased migration of mesenchymal stem cells via CB2 activation and GPR55 antagonism, which resulted in osteoblastic differentiation [24]. The effects of GPR55 on bone may be sex-specific as male GPR55^−/−^ mice, but not female GPR55^−/−^ mice, had more inactive osteoclasts and increased trabecular bone volume and thickness [23].

### Effects of Phytocannabinoids on Bone in Preclinical Studies

CBD was shown to improve bone quality in OVX mice when administered as an early intervention. In C57BL/6 J mice, CBD (25 mg/kg/day, perorally administered 5 days/week for 18 weeks) restored OVX-induced reductions in whole-body and femoral areal BMD (aBMD), improved trabecular quality, and improved markers of bone metabolism [25]. In Sprague–Dawley rats, CBD supplementation (5 mg/kg/day, administered 13 weeks after surgeries via IP injection (5 days/week for 3 weeks) improved femoral BV/TV% (bone volume/total volume of bone) and Tb.N (mean trabecular number), as well as maximum force in femurs and vertebrae, but there was no difference in femoral BMD or expression of receptor activator of nuclear factor kappa-Β ligand (RANKL) and osteoprotegerin (OPG) between the CBD and vehicle treated groups [26]. In contrast, CBD treatment administered by osmotic pump (5 mg/kg/day for 12 weeks) did not restore OVX-induced trabecular bone loss or reduce circulating markers of bone resorption in skeletally mature Sprague–Dawley rats [27]. Differences in the outcomes of these studies may be due to differences in route of CBD administration and potential for interaction of phytocannabinoids and the gut microbiome, as well as age at time of ovariectomy, i.e., during growth vs. maturity. Orally consumed CBD is likely biotransformed by gut bacteria [28] and the effects of the resulting microbial metabolites on bone and other tissues remain to be investigated. Additional research is required to determine the optimal timing and dosage of CBD for mitigating OVX-induced bone loss.

Several rodent studies have shown that CBD may promote bone healing, while other phytocannabinoids are effective for pain management but have mixed effects on bone. When the effects of OVX and CBD on femoral fracture healing were examined C57BL/6 J mice, CBD treatment (5 mg/kg, administered via osmotic pump) that was initiated prior to, but not at the time of the fracture, reduced OVX-induced delayed bone healing [29]. CBD pretreatment did not enhance bone quality or strength in healthy male mice with femoral fractures, but restored fluoxetine-induced reductions in BMD [29]. In a surgical model of spinal cord injury in male Wistar rats, CBD (5 mg/kg/day) administered by IP injections starting 12 h post-surgery for 14 days resulted in increased osteocalcin and reduced collagen type I cross-linked telopeptide in serum, and improved femoral BMD, trabecular quality, and mechanical properties [30]. In male Wistar rats that underwent radial bone ostectomy, treatment with an osteoconductive scaffold containing CBD-loaded microspheres promoted new bone formation, mesenchymal stem cell migration, and increased expression of osteogenic markers compared to control CBD-free scaffold [31]. In a mouse model of collagen-induced arthritis, male DBA/1 J mice that received subcutaneous injections of Δ−8-THC (30 mg/kg, twice per day for 2 weeks) had reduced pain, ankle joint inflammation, and bone erosion [32]. Very few studies have directly compared the effects of different phytocannabinoids which are expected to have divergent effects on bone due to their distinct chemical structures and receptor binding profiles. In female Sprague–Dawley rats with lumbar fusion, histological analysis showed CBD treatment (IP injections of 5 mg/kg/week for 8 weeks) improved bone healing and new bone formation [33]. Osteogenic factors were upregulated after 2 weeks but not 8 weeks, which suggests CBD enhances bone formation in the early stages of healing [33]. In contrast, bone healing was unchanged in rats receiving THC alone or a combination of CBD and THC [33]. In male Sprague–Dawley rats, CBD but not THC treatment (IP injections of 5 mg/kg/day for 2, 4, 6, or 8 weeks) improved maximal load and work-to-failure [34]. CBD increased *Plod1* (lysyl hydroxylase 1) mRNA expression, an enzyme required for collagen synthesis, in primary osteoblast cultures, and may enhance collagen crosslinking [34]. The effect of THC and CBD osteoclast fusion and bone resorption in human osteoclasts was shown to be dose dependent. At lower doses (0.3 to 10 µM), THC and CBD increased bone resorption, but osteoclast fusion was unaffected while at higher doses (10 and 30 µM) osteoclast fusion and bone resorption were inhibited [35]. In vitro studies demonstrated that CBD may support bone regeneration by increasing osteogenic differentiation and reducing osteoclast activity. CBD promoted osteoblast differentiation through p38 MAPK activation in U2OS and MG-63 cells [36], increased cell viability and proliferation and osteocalcin expression in human skeletal stem and progenitor cells (SSPCs) [29], and suppressed tumor-induced osteoclastogenesis [37].

Phytocannabinoids show promise as alternatives to NSAIDs and opioids for fracture pain management; however, their effects during fracture healing are insufficiently investigated. A mouse model of tibial fracture was used to compare the analgesic effects of CBD, CBG, and CBC [38, 39]. CBD and CBG increased bone volume fraction, BMD, and strength and decreased pain [38] while CBC was beneficial for pain management but inhibited bone repair [39].

Studies have investigated CBD in rodent models of periodontitis and suggest phytocannabinoids may support periodontal health. CBD administered by IP injection (5 mg/kg/day for 30 days) attenuated periodontitis-induced alveolar bone loss, inhibited RANK/RANKL expression, and reduced markers of inflammation in male Wistar rats [40]. In male Sprague–Dawley rats, oral treatment with CBD (20 mg/kg/d) in combination with taurine (100 mg/kg) for 21-days reduced periodontal inflammation, pocket depth, and alveolar bone loss [41]. Topically applied CBD (100 mg/kg/day for 4 weeks) inhibited TLR4/NFkB signaling to prevent bone loss and periodontitis-induced inflammation in male Sprague Dawley rats [42]. In vitro studies demonstrated that CBD may protect against alveolar bone resorption by promoting osteogenic differentiation. In human tori-derived bone stromal cells, ex vivo treatment with CBD enhanced osteogenic differentiation, biomineralization, and increased mRNA expression of runt-related transcription factor 2 (RUNX2), bone sialoprotein (BSP), and Osterix [43]. Pretreatment with an AKT inhibitor or β-catenin antagonist reduced these changes, suggesting AKT/β-catenin may be required for CBD to improve osteogenesis [43]. Dental pulp stem cells (DPSCs) are an emerging therapeutic in regenerative dentistry, and recent findings suggest CBD can enhance DPSC function [44–46]. CBD increased DPSC viability, migration, and osteogenic/odontogenic differentiation, and had a pro-angiogenic and anti-inflammatory effect [45]. CBD upregulated osteogenic differentiation and expression of osteogenic markers in DPSC-derived organoid-like microspheroids which promoted bone healing in a nude mouse calvarial defect model [46]. The effect of other phytocannabinoids on bone health remains to be investigated. There has only been one study which demonstrated THC may be beneficial for preventing alveolar bone loss [47], but no studies of other minor cannabinoids. Periodontal width narrowing and bone resorption caused by tooth movement was reversed by treatment with dronabinol, a synthetic THC (10 mg/kg/day for 21 days by IP injection) [47].

Most of the studies investigating the effects of phytocannabinoids on bone have used isolated CBD and there is limited research using extracts containing multiple phytocannabinoids. In a study that used destabilization of the medial meniscus (DMM) surgery to stimulate osteoarthritis in male C57BL/6 J mice, CBD (20 mg/kg/day) and CBD + CBG (10 mg CBD and 10 mg CBG/kg/day) oils reduced pain and inflammation and normalized locomotor activity but did not affect bone remodeling [48]. DMM mice treated with CBG, but not CBD, demonstrated reduced cartilage degeneration, chondrocyte loss, and matrix metalloproteinase-13 expression, suggesting CBG oil is chondroprotective [48].

There is limited data on the effect of cannabis on bone. Male Wistar rats with tibial titanium implants that were forced to inhale cannabis smoke for 8 min per day for 60 days showed decreased trabecular bone healing around the implants, but there was no effect on cortical bone [49]. How bone may be affected by cannabis may depend on factors such as route of administration (smoking, oral, topical, etc.,), age and sex, and hormone status, however this remains to be investigated.

### Effects of Phytocannabinoids on Bone Health in Humans

The rise in cannabis use has generated questions regarding the potential beneficial vs. detrimental effects of different phytocannabinoids on bone health [50, 51] but clinical research is scarce. The United States National Health and Nutrition Examination Survey found no association between cannabis use and BMD in people ages 20–59 years old [52]. Daily supplementation with hemp extract for 6 weeks was found to have no effect on bone mineral content in overweight healthy men and women [53]. In contrast, a cross-sectional study conducted in the UK reported heavy cannabis users had lower total hip and spine BMD, increased fractures, and higher markers of bone turnover compared to cigarette smoking controls [54]. Two clinical studies have demonstrated that CBD and THC may reduce bone resorption [55, 56]. In a case series where 2 postmenopausal women received either 100 or 300 mg CBD daily, orally administered, twice per day for 12 weeks, CBD did not affect sleep disturbance, depression, anxiety, or quality of life but reduced markers of bone turnover in serum, specifically CTx (carboxyl-terminal collagen crosslinks), P1NP (procollagen type 1 N-terminal propeptide), BSAP (bone-specific alkaline phosphatase), and OC (osteocalcin) [55]. Recently, in the first clinical study to test the effects of two medical cannabis products on bone in healthy adults, 38 men and 45 women received softgels containing 5—20 mg THC (with varying CBD levels) twice per day for 7 days. Serum levels of CTx were reduced by both products, but markers of bone formation were unchanged, suggesting phytocannabinoids may enhance bone metabolism [56]. This study was performed in healthy adults, and it is unknown if medical cannabis would have beneficial therapeutic effects in adults who have already experienced bone loss. Further research is needed to better understand how bone may be affected by different phytocannabinoid compounds, dosages, modes of administration, sex differences, and disease status.

## Conclusion

Currently, CBD is the primary phytocannabinoid that has received attention for improving bone health. Though the initial clinical data suggest CBD isolate and medical cannabis may reduce bone turnover, it is unknown if CBD and/or other phytocannabinoids improve bone strength, quality, or BMD in humans. The focus on CBD can partially be attributed to the complex legal status of cannabis. In the United States, the 2018 Farm Bill defined “hemp extract” as containing less than 0.3% delta-9 THC, which removed it from Schedule 1 of the Controlled Substances Act and placed it in Schedule V, making it legal to produce, sell, and transport hemp under federal law. Although widely available and federally legal, FDA does not recognize hemp or hemp-derived isolates such as CBD as a dietary supplement which has led to individual states deciding on how to control the sale of these products. Recreational cannabis remains federally illegal and is a Schedule I controlled substance in the US; however, state licensed medical cannabis and FDA-approved cannabis products are now reclassified as Schedule III. This new classification is expected to facilitate research demonstrating the safety and efficacy of phytocannabinoid preparations in general and for bone health and bone-related disorders.

## Key References

- de Oliveira AC, Macedo AP, Shimano AC. Effects of Cannabidiol on Bone Quality in Ovariectomized Rats. Calcif Tissue Int. 2024;115(5):700–11. [26]
  - ○ This was the first preclinical study to investigate how timing influences CBD’s impact on OVX-induced bone loss. CBD intervention, initiated after the onset of osteopenia, improved bone quality and strength but did not affects markers of bone metabolism.

- Chanpaisaeng K, Fleet JC, Rawiwet V, Phonsatta N, Panya A, Panupinthu N, et al. Long-term cannabidiol treatment did not restore bone microstructural defects in skeletally mature ovariectomized Sprague–Dawley rats. JBMR Plus. 2026;10(2):ziaf197. [27]
  - ○ This study examined the effect of CBD intervention on rats that were OVX after they reached skeletal maturity. CBD treatment did not restore OVX-induced trabecular bone loss or reduce markers of bone resorption in serum.

- Ihejirika-Lomedico R, Patel K, Buchalter DB, Kirby DJ, Mehta D, Dankert JF, et al. Non-psychoactive Cannabidiol Prevents Osteoporosis in an Animal Model and Increases Cell Viability, Proliferation, and Osteogenic Gene Expression in Human Skeletal Stem and Progenitor Cells. Calcif Tissue Int. 2023;112(6):716–26 [29]
  - ○ This study explored the effect of CBD in murine models of femoral fracture and osteoporosis. CBD was most effective for bone healing when used as a preventative treatment.

- Fogel H, Yeritsyan D, Momenzadeh K, Kheir N, Yeung CM, Abbasian M, et al. The effect of cannabinoids on single-level lumbar arthrodesis outcomes in a rat model. Spine J. 2024;24(9):1759–72. [33]
  - ○ This study directly compared the effect of CBD and THC alone and in combination on bone healing. Rats treated with CBD showed the greatest improvements.

- Khajuria DK, Karuppagounder V, Nowak I, Sepulveda DE, Lewis GS, Norbury CC, et al. Cannabidiol and Cannabigerol, Nonpsychotropic Cannabinoids, as Analgesics that Effectively Manage Bone Fracture Pain and Promote Healing in Mice. J Bone Miner Res. 2023;38(11):1560–76 [38]
  - ○ This study demonstrated that CBD and CBG promoted healing and reduced pain in a mose model of tibial fracture.

- Khajuria DK, Wee H, Koroneos ZA, Tantawy MA, Lewis GS, Raup-Konsavage WM, et al. Cannabichromene attenuates fracture pain but impairs bone repair in a murine tibial fracture model. Bone. 2026;207:117,850. [39]
  - ○ This study showed CBC inhibited fracture healing in a mouse model of tibial fracture.

- Kulpa J, Eglit G, Hill ML, MacNair L, Yardley H, Ware MA, et al. Serum Markers of Bone Turnover Following Controlled Administration of Two Medical Cannabis Products in Healthy Adults. Cannabis Cannabinoid Res. 2024;9(1):300–9 [56]
  - ○ This was the first clinical study to test how medical cannabis affects markers of bone turnover and formation in healthy adults.

## Data Availability

No datasets were generated or analysed during the current study.
